# Family network and household composition: a longitudinal dataset derived from the Karonga Health and Demographic Surveillance System, in rural Malawi

**DOI:** 10.12688/wellcomeopenres.20406.1

**Published:** 2023-12-14

**Authors:** Estelle McLean, Fredrick Kalobekamo, Oddie Mwiba, Amelia C Crampin, Emma Slaymaker, Rebecca Sear, Albert Dube

**Affiliations:** 1 Department of Population Health, London School of Hygiene and Tropical Medicine, London, WC1E 7HT, UK; 2 Malawi Epidemiology and Intervention Research Unit, Chilumba, Karonga District, Malawi

**Keywords:** Family, Relatives, Household, GPS, Longitudinal, Malawi

## Abstract

Proximity to family, household composition, and structure are often studied as outcomes and as explanatory factors in a wide range of scientific disciplines. Here, we describe a large longitudinal dataset (currently including data from over 70,000 individuals from 2004 to 2017), including data on household structure, proximity to kin, population density, and other socio-demographic factors derived from data from the Karonga Health and Demographic Surveillance Site (HDSS) in Northern Malawi. We present how the dataset is generated, list some examples of how it can be used, and provide information on the limitations that affect the types of analyses that can be carried out.

## Introduction

Proximity to family, household composition, and structure have been studied and described as outcomes themselves ( Keilman, 1988) and as explanatory factors in a diverse range of disciplines, including nutrition ( Bronte-Tinkew & Dejong, 2004), childhood vaccination ( Gage *et al.*, 1997), poverty ( Snyder *et al.*, 2006), education ( Perkins, 2019), evolutionary biology ( Flinn *et al.*, 2007), criminology ( Maxfield, 1987), child abuse ( Stiffman *et al.*, 2002), transportation ( Strathman *et al.*, 1994) and tourism ( Tangeland & Aas, 2011). This data note describes a large longitudinal dataset (currently including data from over 70,000 individuals from 2004 to 2017), including data on household structure, proximity to kin, population density, and other socio-demographic factors derived from data from the Karonga Health and Demographic Surveillance Site (HDSS) in Northern Malawi. The Karonga HDSS is run by the Malawi Epidemiology and Intervention Research Unit (MEIRU), formerly known as the Karonga Prevention Study. It has been running since 2002 but built upon research infrastructure that has been ongoing in the same area since 1979 ( Ponnighaus *et al.*, 1987). Early research in the area focused on leprosy, and as the disease was known to cluster in families, considerable effort was expended on linking research participants (with and without leprosy) to their parents to generate family lineages. This practice has continued to the present day, allowing the generation of this rich dataset.

## Methods

### Context

The Karonga HDSS was established in 2002 in the southern Karonga district of northern Malawi ( Crampin *et al.*, 2012). The area is largely rural, with one semi-urban trading town, several smaller market villages, and one port in Lake Malawi. The majority of the population engage in subsistence farming or fishing. The main ethnic group living here is Tumbuka, who since the 19 ^th^ century have followed patrilineal and patrilocal customs: women tend to move to their husbands’ villages when they marry ( Malawi Human Rights Commission, 2006). In the event of divorce or even paternal death, children who are old enough to be away from their mothers may be required to live with their fathers’ families ( Malawi Human Rights Commission, 2006). Polygyny is widespread; at the end of 2016, about 15% of households in the HDSS were headed by men with more than one wife.

### Initial data

The HDSS covers an area of 150km ^2^ and by 2016, over 40,000 people were under surveillance. Births and deaths are captured monthly through a system of local ‘key informants,’ whereas migrations are captured annually through visits to all households. Specific dates for each event are captured; therefore, the data are arranged as episodes that may start with the initial census, birth, or in-migration and end with death or out-migration. Participants are given a unique identifier (ID) that they retain in all studies: if they move, they are linked back to this ID (even if they left the area and then returned). Households are also given unique identifiers and the household ID is listed as part of each residency episode. If a participant moves to a new household within the area, the episode at the old household ends, and a new one begins. In the HDSS, a household is defined as a group of individuals, rather than a location, meaning that if the group moves, they would still be classified as the same household. Household membership is defined by the participants under the guidance of trained fieldworkers: all household members must usually live in the dwelling/compound together and recognize the same household head ( Crampin *et al.*, 2012). Men with more than one wife who do not live in the same location are assigned to live in all the co-wives’ households; all other participants may only belong to one household. GPS coordinates were recorded for each household at the initial census, when the household is established, and if it moves. House move or change in household membership may result in one household being ‘dissolved’ and other(s) established. Because the household ID is listed with each person’s residency episode, it is possible to link all individual household members at any time point.

When a new HDSS participant is registered through birth or in-migration, where possible, members of any age are linked to their parents’ identification numbers if they have ever been assigned one. On an annual basis, participants are asked about their marital status and to provide information about their spouse(s), where possible, the identification numbers of the spouses have also been linked. Parents and spouses do not need to be HDSS members themselves to receive an identifying number.

Regular and one-off surveys have been carried out in the area using the HDSS as a platform. Individual and household socio-economic status variables are regularly gathered.

### Data processing

Raw data are currently stored in Microsoft Access databases and extracted in the Stata format. All data processing to create this dataset described in this paper was performed using Stata 16.1.

The longitudinal dataset described in this paper is in the format of an unbalanced panel dataset, with HDSS residents contributing one record for each period while they were living in the HDSS area from 2004 to 2017. The residency episodes are first reduced to one record per person per period by taking a snapshot at the midpoint of the period. This allows for more flexible data manipulation. As continuous data are available for all HDSS residents, the length of the period represented by the snapshot can vary according to the needs of the analysis ( *i.e.*, yearly, quarterly, or monthly). This description uses a mid-year snapshot as an example, but the same processes can be used for any period.

Separately, the parent ID and spouse-ID lists are combined to generate a long list of all blood and non-blood relationships between all HDSS residents. Each relationship record includes the detailed relation type (e.g., mother, half-sister, great-aunt), family type: maternal (mother and any relatives through her [ *i.e.* grandparents, aunts/uncles and cousins), paternal (father and any relatives through him), sister (half or full sister and any of her children or grandchildren), brother (half or full brother and any of his children or grandchildren), daughter (daughter and any of her children or grandchildren), son (son and any of his children or grandchildren), the estimated genetic relatedness ( *i.e.*, 50% for parent-child, down to 3.125% for mother’s cousin), categorized age difference and sex of the relation. For blood relationships, the most distant included were children of cousins and mother’s or father’s cousin; for non-blood relatives, step-family was included up to step-great grandparent/child (though not other step-relations *i.e.,* step-cousins or aunts), spouse, spouse’s family (in-laws), and spouses of blood relatives, both up to cousins/great-grandparents. Being related in more than one way is possible in this area; for example, a widow may marry her deceased husband’s brother, so for her children from the first marriage, the new husband would be both their uncle and step-father. One ‘closest’ relationship was selected as the main one by preferring blood over non-blood relationships and, within the blood relatives, choosing the one with the highest average genetic relatedness. The full list of relations for people with more than one link is also available.

The population panel and the relationships dataset are used in three linked processes that generate variables describing household characteristics, the relationships between the index person and their other household members, and their family network beyond the household. The resulting datasets from the three processes are merged so that all of the above information is available for each person, at each time point that they are present in the HDSS.

### Household characteristics

The population panel data were used to create a summary dataset describing the households at each time point. All households in each mid-year snapshot were first summarized into the number of household members by age group. The age composition of households can be used as an indicator of vulnerability, *i.e.* by calculating the number of working-age adults to dependent children and older adults. Second, the average relatedness between all household members is calculated, which is a measure of kinship within social groups often used in social biology ( Koster, 2018). Finally, the proportion of all the relationships in the household that are unknown is calculated, which is when there is no known blood or non-blood relationship between them, but either one lacks at least one parental ID, so we cannot be sure that they are non-relatives. This is an indicator of the data quality.

The distance between each index household and every other household in the local area is then calculated. The summary household variables were then used to calculate, for each household at each time point, the number of other households, the number of other people (overall and by age group), the mean household relatedness, and the mean level of missing data within certain radii ( *i.e.* 25m, or 250m). These are indicators of population density, several different radiuses are used to reflect the types of habitation that the HDSS covers, to be able to differentiate between households living in the dense trading centre (high density in both narrow and wider radius), in small, isolated clusters of households (high density in narrow radius, but low in wider radius) or in loosely connected villages (medium density in both narrow and wide radius). The population density variables were also used to identify linked households in the analyses (see below).

### Relationships within households

While people in Karonga mostly do not live in shared compounds, as is common in other settings, it was known from field worker reports and through interrogation of the data that two or more households sometimes reside in very close proximity, sharing facilities in loose economic or social alliances, with shared resources and linked prospects. Using the population panel and relationship data, these grouped households were identified to generate an ‘expanded household’ definition. Grouped households were not formally identified during surveillance; thus, a data-driven approach was used to harness the spacing between households at different population densities together with relationship data.

Initially, a random sample of 100 pairs of households 30m or less (but over 0m) apart was examined individually using satellite imagery on Google Earth and assigned by eye as the same or different compounds. The ‘same’ compound households were a median of 7.7m (range 1.7–21.2m, IQR 4.1–11.4) apart while the ‘different’ ones were 18m (range 6–29.5m, IQR 13.7–21.3) apart. Thus, it was assumed that all households less than 5m apart were linked and may be linked if they were up to 20m apart. Individual households were assigned a ‘starting’ radius of five, 10, 15 or 20 metres if the number of households within the radius was more than would be expected given the household density within 50m: a household in a more densely populated area would therefore have a smaller starting radius *i.e.* a household with 20 households within 50m (7852 m ^2^) would expect to have 0.8 within 10m (314.2 m ^2^) and 3.2 within 20m (1256.6 m2) if they are distributed equally, so if they have two households within 10m and three within 20m the initial radius would be set at 10m. If a household had the expected number of households according to the 50m radius it was given a starting radius of 5m. Using the radius as a guide, households were linked if there was at least one relationship link between the households ( *i.e.,* at least one member of one household is related by blood or marriage to at least one member of the other household); not all households within the radius may be grouped if there is a bigger difference between them and the existing cluster. This method is prone to error but results in more appropriate connections between households than using a simpler rule such as all households within 5m (which would reduce the number of connections made in more rural areas where linked buildings can be more spaced out) or within 20m (which would inappropriately connect multiple households in more densely populated areas).

Once all members of each individual’s ‘immediate’ (as recorded in the data) and ‘expanded’ (as described above) households were identified, the listing of all blood and non-blood relationships was used to create binary or continuous variables indicating the presence of certain relative types, *i.e.* mother in immediate household, or number of maternal half siblings aged under 18 in expanded households.

### Family network

The GPS coordinates of all blood relatives (either singly or as groups, *i.e.*, maternal or paternal) were compared to those of the index at each time point. Summary variables were then calculated as either binary or continuous for the presence of relatives within certain radii (e.g., father living with 250m, number of maternal aunts aged over 18 living within 100m, number of paternal relatives living within 50m). These variables are named and coded similarly to household-relative variables.

## Examples of uses of dataset

This dataset has been used in an in-depth analysis of household composition, including an assessment of whether latent class analysis can be used to create data-driven household classifications ( McLean *et al.*, 2021a), an analysis of transition to adulthood by using household composition variables to identify when an adolescent can be described as having left home (along with other variables related to leaving school, getting married, and having children) in a sequence analysis ( McLean *et al.*, 2021b) and an analysis of the effect of the presence of family within and outside the household on short and long migration in children and adolescents ( McLean *et al.*, 2023). Other analyses related to mortality and fertility are possible, and as the HDSS is ongoing, more analyses linking childhood household composition/structure with adult outcomes will be possible. Newly collected data can be added to the datasets by re-running all the processes with the updated datasets. Other HDSSs collect similar data, and thus may be able to generate similar datasets, following the logic described above.

## Dataset validation / limitations

**Figure 1.**
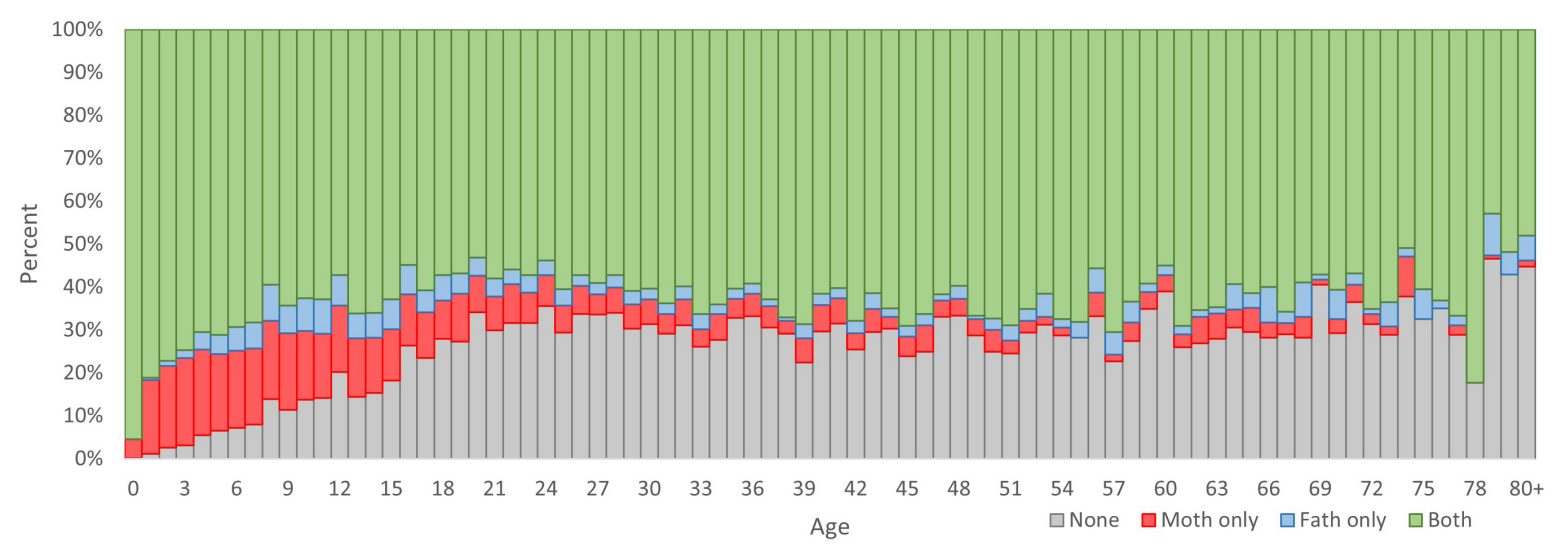
Percent of HDSS residents by age and availability of parent ID-links. Percent of participants with no parent ID-links are at the bottom of the columns in grey, above that in red are those with only mother ID-link, above that in blue are those with only father ID-link, and at the top in green are those with both parent ID-links.

Although this dataset has many potential uses, it is important for users to be aware of some limitations to aid in the appropriate selection of data for analyses. The dataset is dependent on parent and spouse links, which are not available for all HDSS members. The proportion of all HDSS members by age and whether their mother and father IDs are known is shown in Figure 1. Children had the highest proportion of known parental IDS, and there was very good coverage for the youngest children. After childhood, the proportion with no ids is relatively stable at around 30%, with most people having both mother and father ID available.

**Figure 2.**
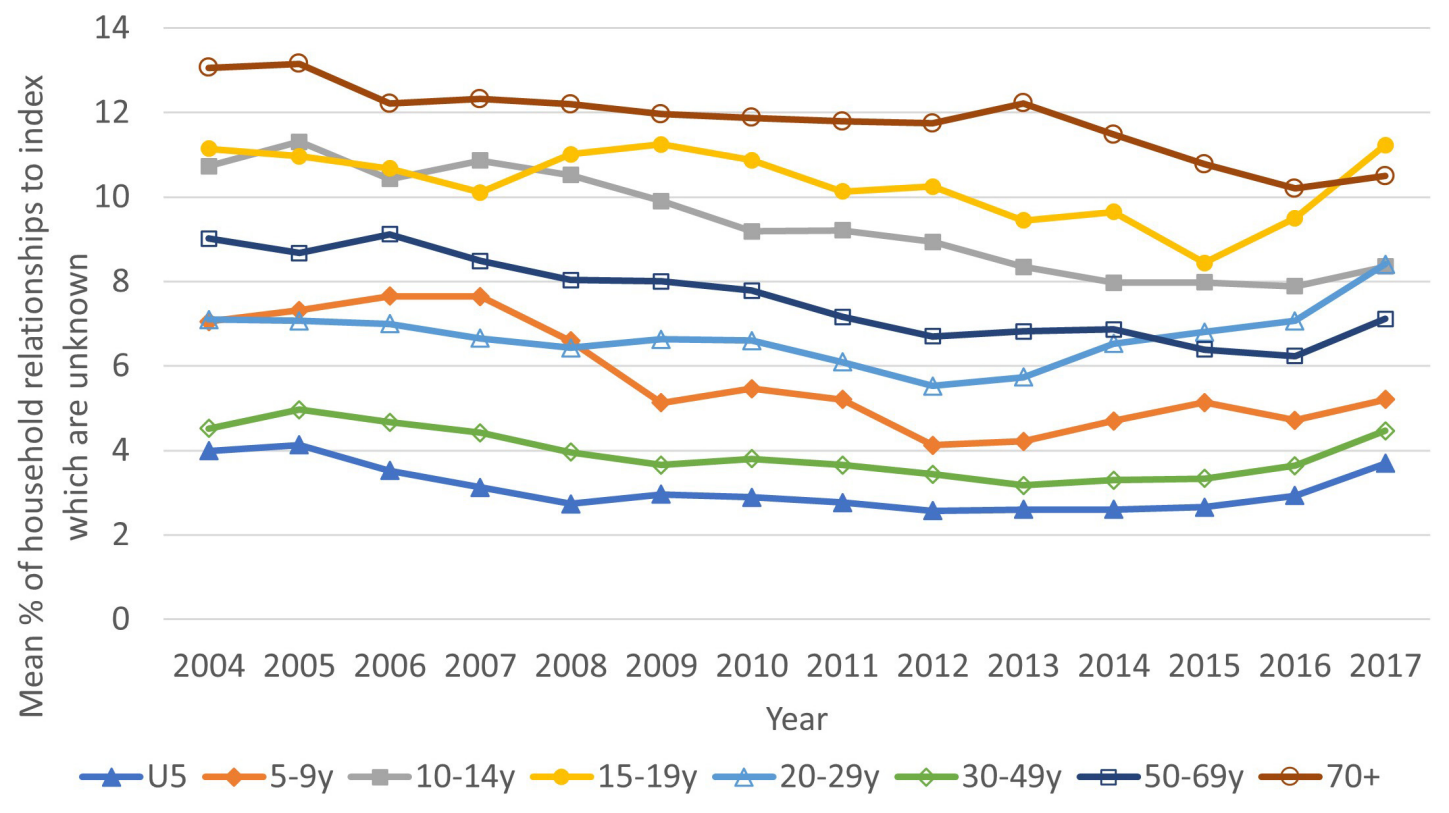
Average percent of relationships to index person within households which are unknown, by age group (of index) and year. Data for under-fives is shown with solid blue triangles, for five-nine year-olds with solid orange diamonds, for 10–14 year-olds with solid grey squares, for 15–19 year-olds with empty yellow circles, for 20–29 year-olds with empty blue triangles, for 30–39 year-olds with empty green diamonds, for 50–59 year-olds with empty dark blue squares and for 70+ with empty maroon circles.

Being able to link individuals to their relatives also depends on whether other people have parent/spouse ID links. Figure 2 shows the average proportion of household relationships unknown by the age of the index person and calendar year. Unsurprisingly, the group with the lowest proportion of unknown relationships is children aged under five, but the 30–49-year age group also has low levels (as their households are likely to be formed of their spouse and children). The groups with the highest proportion of unknown relationships were people aged over 70 years and adolescents aged 15–19, however the proportions were not high (under 13%). By calendar year, the proportions unknown decreased somewhat from 2004, but there was an increase at the end of the period to 2017.

The actual number of individuals available in the dataset by year and age group is shown in Table 1, which also shows the proportion with complete information on their relationships with all household members and the proportion with no information at all. This shows that there are a high number of individuals with sufficient data in all age groups, although the numbers decrease after the age of 50 years.

**Table 1.** Total number of individuals* in the dataset by age group, selected years and whether their relationship to other household members are fully known or fully unknown.

Age group	Households		2005	2007	2009	2011	2013	2015	2017
All	Total	n	31596	33685	34027	35833	37787	40453	43523
	Fully known	n	25112	27343	28250	30169	32272	34334	35864
		%	79.5%	81.2%	83.0%	84.2%	85.4%	84.9%	82.4%
	Fully unknown	n	1017	1062	1025	1019	1025	1134	1488
		%	3.2%	3.2%	3.0%	2.8%	2.7%	2.8%	3.4%
U5	Total	n	6161	6671	6437	6350	6265	6409	6311
	Fully known	n	5281	5886	5762	5738	5729	5852	5607
		%	85.7%	88.2%	89.5%	90.4%	91.4%	91.3%	88.8%
	Fully unknown	n	60	37	42	34	43	42	64
		%	1.0%	0.6%	0.7%	0.5%	0.7%	0.7%	1.0%
5–9y	Total	n	4696	5424	5315	6033	6230	6473	6565
	Fully known	n	3831	4421	4554	5217	5512	5661	5659
		%	81.6%	81.5%	85.7%	86.5%	88.5%	87.5%	86.2%
	Fully unknown	n	188	231	128	159	128	183	168
		%	4.0%	4.3%	2.4%	2.6%	2.1%	2.8%	2.6%
10–14y	Total	n	4107	4297	4418	4847	4979	5852	6631
	Fully known	n	3121	3326	3520	3959	4138	4880	5442
		%	76.0%	77.4%	79.7%	81.7%	83.1%	83.4%	82.1%
	Fully unknown	n	306	317	298	300	281	310	354
		%	7.5%	7.4%	6.7%	6.2%	5.6%	5.3%	5.3%
15–19y	Total	n	2943	2883	3473	3435	4115	4288	4818
	Fully known	n	2208	2241	2730	2740	3389	3536	3757
		%	75.0%	77.7%	78.6%	79.8%	82.4%	82.5%	78.0%
	Fully unknown	n	187	177	268	234	277	241	371
		%	6.4%	6.1%	7.7%	6.8%	6.7%	5.6%	7.7%
20–29y	Total	n	5315	5775	5076	5529	5537	6266	6889
	Fully known	n	4261	4733	4209	4687	4753	5250	5560
		%	80.2%	82.0%	82.9%	84.8%	85.8%	83.8%	80.7%
	Fully unknown	n	145	176	159	174	170	237	329
		%	2.7%	3.0%	3.1%	3.1%	3.1%	3.8%	4.8%
30–49y	Total	n	5202	5514	5943	6273	6966	7340	8045
	Fully known	n	4239	4568	5084	5389	6044	6336	6746
		%	81.5%	82.8%	85.5%	85.9%	86.8%	86.3%	83.9%
	Fully unknown	n	55	49	44	48	40	48	106
		%	1.1%	0.9%	0.7%	0.8%	0.6%	0.7%	1.3%
50–69y	Total	n	2173	2128	2328	2376	2605	2805	3038
	Fully known	n	1558	1527	1713	1799	1999	2151	2288
		%	71.7%	71.8%	73.6%	75.7%	76.7%	76.7%	75.3%
	Fully unknown	n	42	36	46	35	42	38	57
		%	1.9%	1.7%	2.0%	1.5%	1.6%	1.4%	1.9%
70+	Total	n	999	993	1037	990	1090	1020	1226
	Fully known	n	613	641	678	640	708	668	805
		%	61.4%	64.6%	65.4%	64.6%	65.0%	65.5%	65.7%
	Fully unknown	n	34	39	40	35	44	35	39
		%	3.4%	3.9%	3.9%	3.5%	4.0%	3.4%	3.2%

Note that individuals contribute data to this table for all years which they are present in the HDSS.

Another potential limitation of the dataset is related to the HDSS data source: data are only available on participants when they live in the HDSS area. Table 2 shows the number of HDSS residents by sex and birth cohort and the number of years they were present in the HDSS between 2004 and 2017 (maximum 14 years). While a large number of participants have complete data for the whole 14-year period, it is important to note that those who remain in the area are likely to be different from those who do not. Table 2 shows the effects of birth cohort (those with earlier birth dates are more likely to have complete data) and sex (males are more likely to have complete data).

**Table 2.** Number of HDSS residents by age and sex, and how many years they were present.

Sex & birth cohort		Years present in the HDSS							
		1–2y	3–4y	5–6y	7–8y	9–10y	11–12y	13–14y	Total
*Male*									
pre-1960	n	303	192	145	137	108	125	961	1,971
	%	15.4%	9.7%	7.4%	7.0%	5.5%	6.3%	48.8%	
1960–69	n	277	179	121	102	87	91	691	1,548
	%	17.9%	11.6%	7.8%	6.6%	5.6%	5.9%	44.6%	
1979–79	n	662	376	232	208	167	191	1119	2,955
	%	22.4%	12.7%	7.9%	7.0%	5.7%	6.5%	37.9%	
1980–89	n	1167	698	453	380	370	355	1263	4,686
	%	24.9%	14.9%	9.7%	8.1%	7.9%	7.6%	27.0%	
1990–99	n	1327	756	518	442	457	567	2459	6,526
	%	20.3%	11.6%	7.9%	6.8%	7.0%	8.7%	37.7%	
2000–9	n	1909	1024	769	914	1342	1291	2191	9,440
	%	20.2%	10.8%	8.1%	9.7%	14.2%	13.7%	23.2%	
post-2010	n	2201	1576	1145	739	0	0	0	5,661
	%	38.9%	27.8%	20.2%	13.1%	0.0%	0.0%	0.0%	
Total	n	7846	4801	3383	2922	2531	2620	8684	32,787
	%	23.9%	14.6%	10.3%	8.9%	7.7%	8.0%	26.5%	
*Female*									
pre-1960	n	328	263	170	145	162	167	1369	2,604
	%	12.6%	10.1%	6.5%	5.6%	6.2%	6.4%	52.6%	
1960–69	n	232	145	111	100	73	83	878	1,622
	%	14.3%	8.9%	6.8%	6.2%	4.5%	5.1%	54.1%	
1979–79	n	630	393	258	224	193	242	1269	3,209
	%	19.6%	12.2%	8.0%	7.0%	6.0%	7.5%	39.5%	
1980–89	n	1556	869	606	455	427	450	1317	5,680
	%	27.4%	15.3%	10.7%	8.0%	7.5%	7.9%	23.2%	
1990–99	n	2399	1328	978	769	695	751	1648	8,568
	%	28.0%	15.5%	11.4%	9.0%	8.1%	8.8%	19.2%	
2000–9	n	2352	1187	879	956	1473	1327	1787	9,961
	%	23.6%	11.9%	8.8%	9.6%	14.8%	13.3%	17.9%	
post-2010	n	2243	1591	1197	684	0	0	0	5,715
	%	39.2%	27.8%	20.9%	12.0%	0.0%	0.0%	0.0%	
Total	n	9740	5776	4199	3333	3023	3020	8268	37,359
	%	26.1%	15.5%	11.2%	8.9%	8.1%	8.1%	22.1%	

## Ethics

The Karonga HDSS has ethical approval from the outset of the Malawi National Health Science Review Committee (approval #20/11/2641, previously #416) and the London School of Hygiene and Tropical Medicine (approval #5081). All households provided written consent to participate in the Karonga HDSS, which could be rescinded at any time.

## Data Availability

Due to the detailed nature of the data describing the exact living arrangement of participants, it is not possible to anonymize it sufficiently in a way that allows it to still be useful; thus, the data are not available for open access. However, MEIRU welcomes requests to use data from bona fide researchers who should contact the first author (EM) in the first instance. Information of MEIRU datasets can be found on the MEIRU website. Code is available through Zenodo: Family network and household composition: a longitudinal dataset derived from the Karonga HDSS, in rural Malawi (author-written code) https://zenodo.org/records/10037084 This project contains the following files: 0_master_KarongaHDSS_household_family.do: A Stata do-file which calls the following processing do-files. 1_identify_relatives.do: A Stata do-file in which all relative pairs are identified from parent and spouse id linkage lists for use in later do-files. 2_create_snapshots.do: A Stata do-file in which continuous HDSS residency episode data are reduced to snapshots. 3_assign_gps_to_snapshots.do: A Stata do-file in which GPS coordinates are added for each person for the household they are living in in each snapshot. 4_popdens_assign_hhmemb.do: A Stata do-file in which household summary variables are created, including population density and average relatedness within households. 5_id_household_members.do: A Stata do-file in which relationship between index and all household members are identified and summary variables created. 6_add_rels_10_250m: A Stata do-file in which index person's relatives within certain distances are identified and summary variables created. 7_get_other_datasets_ready.do: A Stata do-file in which other datasets related to socio-economic status and other factors are prepared for merging to the main dataset. 8_add_person_hh_states.do: A Stata do-file in which other datasets created in do-file 7 are merged to the main dataset and summary variables created. 9_combine_label_datasets.do: A Stata do-file in which all datasets are combined and labelled ready for use. These files are available under the terms of the Creative Commons Attribution 4.0 International.
