# Supplementary material for: Family network and household composition: a longitudinal dataset derived from the Karonga Health and Demographic Surveillance System, in rural Malawi
Source: Wellcome Open Res. 2024 Apr 30;8:573. Originally published 2023 Dec 14. [Version 2] doi: 10.12688/wellcomeopenres.20406.2 (PMC11148530; doi:10.12688/wellcomeopenres.20406.2)
Supplement: Peer review history

# Reviewer report: Reviewer response for version 2

**DOI:** 10.21956/wellcomeopenres.23727.r81935

**Published:** 2024-06-21

**Authors:** Sarah Reynolds

**Affiliations:** 1 University of California Berkeley, Berkeley, California, USA

Peer Review of “Family network and household composition: a longitudinal dataset derived from the Karonga Health and Demographic Surveillance System, in rural Malawi [version 2]”

 Data Note

- Are sufficient details of methods and materials provided to allow replication by others?

Do files are available by request, so this code could be adapted if others had the raw data.  I have not viewed the do files, so am unable to comment on the clarity of their annotations.

 This is an exciting transformation of an administrative database to provide documentation of the relationships between all household members and their family proximity over time.  Overall this work is thoroughly and clearly presented, and presents the possibility for an exciting expansion of the literature on family composition within and beyond the household longitudinally. I only have a few questions:

- Table 1 has data from every 4 years, Table 2 from every other year, and Figure 3 from every year.  Improve consistency & explain in paper or footnote why these years were selected (except the one with every year)
- With what frequency is the household asset data collected? 
- Understanding attrition is complicated.  An illustration something like Figure 2 could illustrate ages at baseline and what percentage of participants are present at each year, with, of course 100% at baseline.  This suggestion, however, leaves out individuals added at later years.  Perhaps this is too complex to integrate and will be overwhelming – kudos if the author can think of a clever way to incorporate such information!

Highlighting some future directions can help researchers decide to use the data.

- For future applications, what variables link to external data sets?  National ID number, cell-phone number, names? 
- As a future extension, it could be noted that researchers could adjust snapshots to a particular relationship of interest.  For example, if a researcher was interested in mother-child co-residence, the researcher could modify the do-files to focus only on this subset of relationships and create continuous information rather than over 4 years.   
- Similarly, as an alternative to the binary approach for the presence of a relative at the snapshot point, an extension could be the percentage of time over the snapshot period during which the relative was present in the household. 

Are sufficient details of methods and materials provided to allow replication by others?

Yes

Is the rationale for creating the dataset(s) clearly described?

Yes

Are the datasets clearly presented in a useable and accessible format?

Yes

Are the protocols appropriate and is the work technically sound?

Yes

Reviewer Expertise:

Economics, Child Development, Family Demography, Intimate Partner Violence

I confirm that I have read this submission and believe that I have an appropriate level of expertise to confirm that it is of an acceptable scientific standard.

# Reviewer report: Reviewer response for version 2

**DOI:** 10.21956/wellcomeopenres.23727.r83561

**Published:** 2024-06-03

**Authors:** Laurie DeRose

**Affiliations:** 1 The Catholic University of America, Washington, District of Columbia, USA

The theoretical justification is clear: households matter in shaping and reflecting behavior. I get a clear picture of a complex data set. Even where I'm not thrilled with the process (identifying linked households), the immediate household are still preserved.

 I like the way the files of the analysis code are organized.

Are sufficient details of methods and materials provided to allow replication by others?

Yes

Is the rationale for creating the dataset(s) clearly described?

Yes

Are the datasets clearly presented in a useable and accessible format?

Yes

Are the protocols appropriate and is the work technically sound?

Partly

Reviewer Expertise:

Social demography

I confirm that I have read this submission and believe that I have an appropriate level of expertise to confirm that it is of an acceptable scientific standard.

# Reviewer report: Reviewer response for version 2

**DOI:** 10.21956/wellcomeopenres.23727.r81007

**Published:** 2024-05-14

**Authors:** Ashira Menashe-Oren

**Affiliations:** 1 Centre for Demographic Research (DEMO), Universite catholique de Louvain, Louvain-la-Neuve, Walloon Region, Belgium

Thank you for addressing my previous review. I have no further comments.

Are sufficient details of methods and materials provided to allow replication by others?

Partly

Is the rationale for creating the dataset(s) clearly described?

Yes

Are the datasets clearly presented in a useable and accessible format?

Partly

Are the protocols appropriate and is the work technically sound?

Yes

Reviewer Expertise:

Demography

I confirm that I have read this submission and believe that I have an appropriate level of expertise to confirm that it is of an acceptable scientific standard.

# Reviewer report: Reviewer response for version 1

**DOI:** 10.21956/wellcomeopenres.22594.r75859

**Published:** 2024-03-22

**Authors:** Ashira Menashe-Oren

**Affiliations:** 1 Centre for Demographic Research (DEMO), Universite catholique de Louvain, Louvain-la-Neuve, Walloon Region, Belgium

This data note is a useful description of procedures in creating measures of household and kin structures with longitudinal data from a Health and Demographic Surveillance Site (HDSS). It is well-written and justified. I have two issues that I would like to see addressed (and a few minor comments for clarification or suggestions for improvement).

1. The data note informs on the process of generating variables describing household characteristics. It would be helpful if a list of each of these variables is noted up front at the very least. A table of summary statistics of some these variables (household size, genetic relatedness, presence of relatives within radii etc). Mostly, a few tables with examples of these variables seems essential – a couple of households with their members records, and then the various indicators (like one would see in Stata data browser). This would be especially helpful for non-Stata users since the code is only made available in Stata.
2. The section explaining the relationship between households is not clear, in particular the paragraph starting “Initially, a random sample of 100 pairs of households…”. I suggest revising this, and considering adding a flow-chart of the different steps taken.

Some minor comments:

 Justification of using data from 2004 is needed, if the DSS was established in 2002.

 The data is reduced to one record per person per period – taking a snapshot of mid-point. Does this not flatten/ simplify the data unnecessarily? One of the key advantages of the longitudinal data is the timing and order of events.

 The parents or spouses who not reside in DSS but have identifying number – are they included in any of these household measures?

 Is the proportion of unknown relationships truly an indicator of data quality? Is it not feasible for household members to not be related in Karonga?

 Figure 2 could be for grouped years rather than single years.

 Table 2 would be better as a population pyramid.

 The data note ends rather abruptly. It would be nice to see a summary-like paragraph (before ethics section), relating back to the introduction.

Are sufficient details of methods and materials provided to allow replication by others?

Partly

Is the rationale for creating the dataset(s) clearly described?

Yes

Are the datasets clearly presented in a useable and accessible format?

Partly

Are the protocols appropriate and is the work technically sound?

Yes

Reviewer Expertise:

Demography

I confirm that I have read this submission and believe that I have an appropriate level of expertise to confirm that it is of an acceptable scientific standard, however I have significant reservations, as outlined above.

# Response

**Published:** 2024-04-05

**Authors:** Estelle McLean

**Affiliations:** Department of Population Health, London School of Hygiene and Tropical Medicine, UK

Thank you for your very helpful review. For your main 2 points: I have added in a table of some summary statistics, and I have added the link to the full documentation for the dataset which was inadvertently missing (table 1). I have tried to make the description of the methodology used for linking household clearer, and have added a diagram (figure 1). For your minor points: I have clarified in the text that the HDSS was set up from 2002-2004, so 2004 is the first complete year.

   I have clarified the reason for reducing the longitudinal dataset to snapshots – the large number of variables and their rate of change (the combination of relatives living within 250 metres could potentially change very frequently) would mean that splitting the episodes and assigning these variables would be too computationally intense. Absent relatives are not included in this particular dataset, but could be identified if an analysis called for it. Unknown relationships refer to the missing identifiers – non-relatives may be identified if both have parent identifiers but no relationship link is found, I have clarified this in the text. I have not changed figure 2 to used grouped years, as using single years highlights the increase in unknown data in the last few years of the period. I have converted table 2 to a population pyramid figure (figure 4). I have added a summary paragraph before the ethics section.
